# Role of apigenin in targeting metabolic syndrome: A systematic review

**DOI:** 10.22038/IJBMS.2024.71539.15558

**Published:** 2024

**Authors:** Behjat Javadi, Zahra Sobhani

**Affiliations:** 1 Department of Traditional Pharmacy, School of Pharmacy, Mashhad University of Medical Sciences, Mashhad, Iran

**Keywords:** Apigenin, Insulin resistance, Metabolic syndrome, Natural products, Obesity

## Abstract

Metabolic syndrome (MetS) is a cluster of metabolic abnormalities that has a high prevalence worldwide. Apigenin is a flavonoid present in several vegetables and fruits and has anti-inflammatory, anti-oxidant, and anti-MetS properties. This study aims to systematically review the effects of apigenin against MetS and the relevant molecular and cellular mechanisms of action, pharmacokinetics features, and potential structure-activity relationship. Electronic databases including Scopus, PubMed, Science Direct and Cochrane Library were searched for in vivo, and in vitro, and human studies with the following keywords: “apigenin” and “metabolic syndrome or insulin resistance syndrome”, “fatty liver”, “hypertension or blood pressure”, “diabetes or blood glucose”, “dyslipidemia”, “heart or cardiovascular ” and “obesity” in title/abstract. Data were collected from 2000 until 2021 (up to April). Only papers published in the English language were included. Forty-six full-text articles out of 1016 retrieved papers were reviewed and underwent quality assessment by investigators. Anti-obesity activity of apigenin is mainly through attenuating adipocyte differentiation by suppressing the mitotic clonal expansion and the adipogenesis-related factors. Its anti-diabetic effects can be exerted through inhibition of protein tyrosine phosphatase1B expression, maintaining the activity of anti-oxidant enzymes, reducing intracellular ROS production, cellular DNA damage, protein carbonylation, and attenuating β-cell apoptosis. Moreover, apigenin could attenuate dyslipidemia and subsequent atherosclerotic conditions through down-regulating sterol regulatory element-binding proteins (SREBP)-1c, SREBP-2, stearyl-CoA desaturase-1, and 3-hydroxy-3-methyl-glutaryl-CoA reductase. Apigenin as a dietary bioactive compound would be a promising candidate for improving MetS and its components.

## Introduction

Metabolic syndrome (MetS) is defined by WHO as a cluster of metabolic abnormalities characterized by abdominal obesity, insulin resistance (IR), hypertension (HT), and hyperlipidemia (1, 2). Atherogenic dyslipidemia, modulation of nuclear peroxisome proliferator-activated (PPAR), and pro-inflammatory states are also considered the principal components of MetS (3) which is robustly linked to a high risk of developing atherosclerotic cardiovascular disease (CVD) (4). 

MetS prevalence has been shown to be 20% to 45% of the population and is expected to rise to nearly 53% by 2035 (3). This high incidence is strongly associated with the worldwide increase in obesity (4). Lifestyle factors, e.g., excessive food intake and physical inactivity contribute to an increase in oxidative stress (OS) which in turn can result in the development of Type 2 diabetes mellitus (T2DM) by activating stress-signaling pathways, such as the NF-kB pathway as well as decreasing intracellular anti-oxidant defense enzymes (5). 

IR-mediated increase in serum free fatty acids (FFAs) plays a pivotal role in the pathogenesis of MetS (6). Insulin elevates glucose uptake in muscles and liver and suppresses lipolysis and hepatic gluconeogenesis. IR in adipose tissue (AT) diminishes insulin-induced lipolysis inhibition, resulting in an increase in serum FFAs and thus, inhibiting the antilipolytic effect of insulin (7, 8). In obesity, macrophages attack AT and increase the release of cytokines such as interleukin-6 (IL-6) and tumor necrosis factor-alpha (TNF-α), leading to inflammation, atherosclerosis, and vascular endothelial dysfunction. The released cytokines may induce IR in skeletal muscle and change the activity of the pituitary-adrenal axis, leading to the loss of pancreatic beta cells. Moreover, adipokines released from visceral AT are associated with MetS (9). 

The analysis of dietary habits has been used to elucidate the relationship between diet and chronic disease. Natural products have played a key role in the prevention and treatment of various diseases throughout human history. Moreover, they have highly contributed to the discovery and development of modern drugs (10). In particular, medicinal plants and their phytochemicals have been a valuable source of new therapeutic agents and pharmaceuticals for decades. Noteworthy to mention that several natural products and their derivatives were approved by the FDA in 2014. A variety of drugs with herbal origin such as physostigmine from *Physostigma venenosum,* hyoscine from *Hyoscyamus niger*, digitoxin from *Digitalis purpurea,* and ephedrine from *Ephedra sinica* have been developed and prescribed in the Modern era (11-13). Flavonoids, a group of natural compounds with various phenolic structures, are found in all parts of plants, particularly in flowers, fruits, and leaves. Flavonoids or flavonoid-rich foods have been shown to lower the risk of MetS mainly by targeting inflammatory signals (14, 15). 

**Figure 1:**
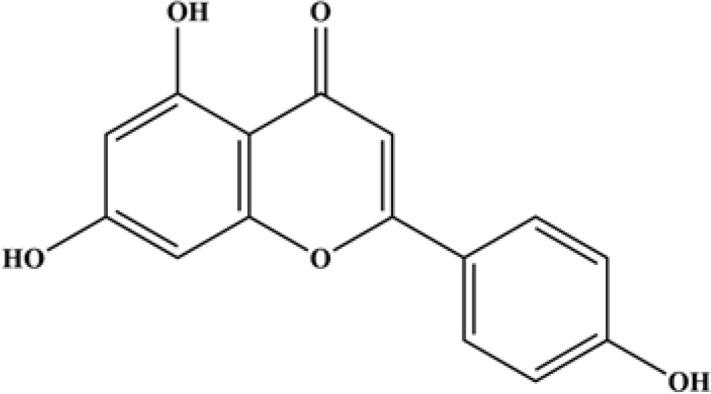
Structure of apigenin

4’,5,7-trihydroxyflavone, commonly known as apigenin (C_15_H_10_O_5_) (Figure 1) is a flavonoid present in several vegetables and fruits such as apiaceous vegetables, citrus fruits, thyme, chamomile, onions, and spices, as well as plant-based beverages (16). Apigenin along with quercetin, kaempferol, myricetin, and luteolin, are the five most common herbal flavonoids (17). It is derived from the phenylpropanoid unit (C6-C3) and is principally present in glycosylated form (17). Apigenin possesses a variety of pharmacological activities such as anticancer (18), anti-inflammatory, and anti-oxidant properties and has been shown to ameliorate MetS. It has low toxicity and no mutagenicity (19-21). This evidence suggests apigenin could be a highly promising candidate for future drug development. 

Given the above, our research aims to systematically review the beneficial effects of apigenin in preventing and attenuating MetS and its components. We also study the relevant molecular and cellular mechanisms of actions of apigenin, its pharmacokinetics features, and potential structure-activity relationship. 

## Materials and Methods

***Information sources/ search strategy***

Electronic databases including Scopus, PubMed, Science Direct, and Cochrane Library were searched for *in vivo*, *in vitro*, and human studies with the keywords “apigenin” and “metabolic syndrome or insulin resistance syndrome ”, “fatty liver”, “hypertension or blood pressure”, “diabetes or blood glucose”, “dyslipidemia”, “heart or cardiovascular ”, and “obesity” in title/abstract. Data were collected from 2000 until 2021 (up to April). Only papers published in English were included.

We also followed PRISMA guidelines while performing the study (http://www.prisma-statement.org/).

***Eligibility criteria***

All original studies and abstracts that investigated the effect of apigenin against various components of MetS in humans, animal (*in vivo*), and cellular (*in vitro*) platforms were included. English language articles were reviewed. Review articles were excluded from the study. However, the reviews were evaluated to ensure that their cited references were included in our initial search.

***Data collection process/ data items***

Two investigators independently screened the titles and abstracts of each eligible study. They further discussed how to resolve disagreements. Data on authors, publication date, setting (*in vivo* or *in vitro*), cellular type, experimented subjects, dosage, time of exposure/number of days, effect sizes, mechanisms, key points, and references were retrieved from the selected reports. 

***Study risk of bias assessment***

Two independent authors evaluated all eligible studies using the following checklists and further discussed to resolve disagreements. *In vivo* animal studies were assessed using Animal Research: Reporting *in vivo* experiments (ARRIVE) checklists applicable items to ensure adequate design and implementation of the animal studies. ARRIVE uses 20 items, each scored as 0 or 1, to summarize the minimum data required for the reporting of animal investigations (22). Random and blinded distribution and allocation of animals to experimental groups, outcome analysis, as well as evaluating the sample size are among the important ARRIVE checklists’ items. The methodological quality of *in vitro* studies was confirmed using OECD GD with Good *in vitro* Method Practices (GIVIMP) (23). 

## Results

***Study selection***

**Figure 2:**
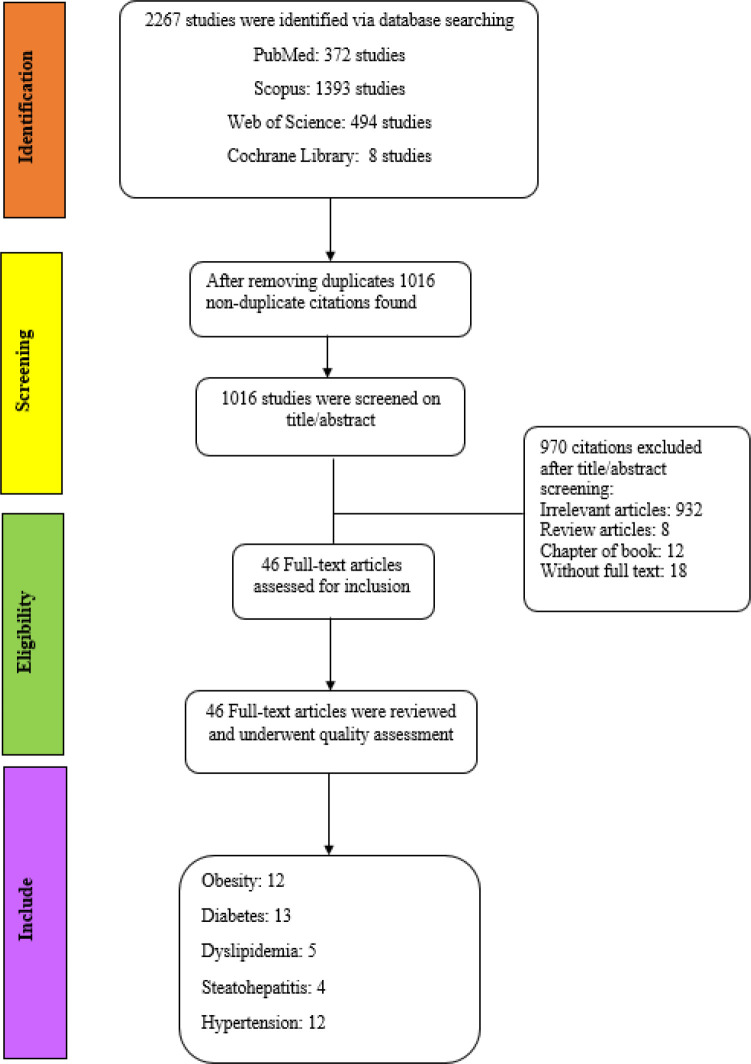
Flow chart: Search strategy and the number of articles from identification to the final inclusion step as well as the exclusion and inclusion criteria

After removing duplicate results, we found a total of 1016 articles in our first search step. Upon evaluating the titles and abstracts, 970 articles were excluded due to unrelated content, book chapters, review articles, and lack of access to the full text of the articles. Finally, 46 papers were reviewed in full, including 12 reports on obesity, 13 reports on diabetes, 5 reports on dyslipidemia, 4 reports on steatohepatitis, and 12 reports on hypertension. The search strategy, the number of articles from identification to the final inclusion, and the studies excluded and included at each step based on the criteria are shown in Figure 2. 

***Study characteristics***

Four *in vivo*, one hybrid (*in vivo* and *in vitro*), and seven *in vitro* studies were evaluated among 12 articles on obesity. Thirteen papers on diabetes included seven *in vivo* and six *in vitro* experiments. There were 12 studies on hypertension, six of which were *in vivo*, one was hybrid (*in vivo* and *in-vitro*), and five were *in vitro*. Three *in vivo*, two *in vitro *were involved in dyslipidemia. Four papers on steatohepatitis included one hybrid and three *in vitro*. No clinical trial or human study investigating the effects of apigenin against MetS has been performed. 

***Pathogenesis of metabolic syndrome***

MetS has a complex pathogenesis, which arises from multiple interacting pathways and genetic and environmental factors such as excess caloric intake and sedentary lifestyles (24). IR, the most prevalent component of MetS, is defined as the inability of the liver, skeletal muscles, AT, etc., to respond to normal levels of insulin. During physiological states, insulin binds to its receptors at the insulin-sensitive sites, which in turn triggers tyrosine phosphorylation and activation of downstream substrate protein cascades including phosphoinositide 3-kinase (PI3K), Akt (protein kinase B), mTOR (molecular target for rapamycin), and Ras-MAPK (mitogen-activated protein kinase) signaling pathways. PI3K activation recruits GLUT4 to transport glucose into muscles and AT. However, during compensatory hyperinsulinemia in IR patients, alterations in insulin secretion or clearance result in mild forms of glucose intolerance, dyslipidemia, and hypertension (24, 25). 

On the other hand, oxidative stress triggers adipocytic IR and induces secretion of leptin, IL-6, and TNF-α by adipocytes. The resulting ROS decreases the secretion of adiponectin in adipocytes. Obesity itself can cause oxidative stress, which in turn stimulates PKC-δ, and activates Nox, causing an increase in O^2^ radical formation. Oxidative stress can induce IR in adipocytes and suppress insulin-dependent activation of PI3K, while the activity of adipocytic JNK and PKC-δ is increased. Impaired PI3K activity and stimulation of PKC-δ in oxidative conditions can directly lead to the formation of the MetS syndrome (26). Overall, AT contributes to the development of obesity-induced inflammation by increasing cytokines and chemokines and dysregulation of adipokines such as TNF-ɑ, IL-6, IL-8, IL-1, monocyte chemoattractant protein-1 (MCP-1), leptin, PAI-1, retinol-binding protein 4 (RBP4), chemerin, serum amyloid A (SSA), C-reactive protein (CRP), lipopolysaccharide-binding protein (LBP), fetuin-A, and decreased adiponectin and omentin-1 (27).

Additionally, systemic inflammation can induce CVD through several immune system biomarkers strongly associated with MetS. Therefore, pro-inflammatory cytokines such as TNF and IL-ß are closely associated with MetS. Chronic inflammation links MetS to IR and CVD via promotion of vascular dysfunction (28). Adipocyte hypertrophy, altered local blood flow, hypoxia, abnormal adipokine expression, and local infiltration of immune cells all contribute to adiposopathy and elevated TNF and IL-6 (25). 

***Effects of apigenin on obesity***

**Figure 3:**
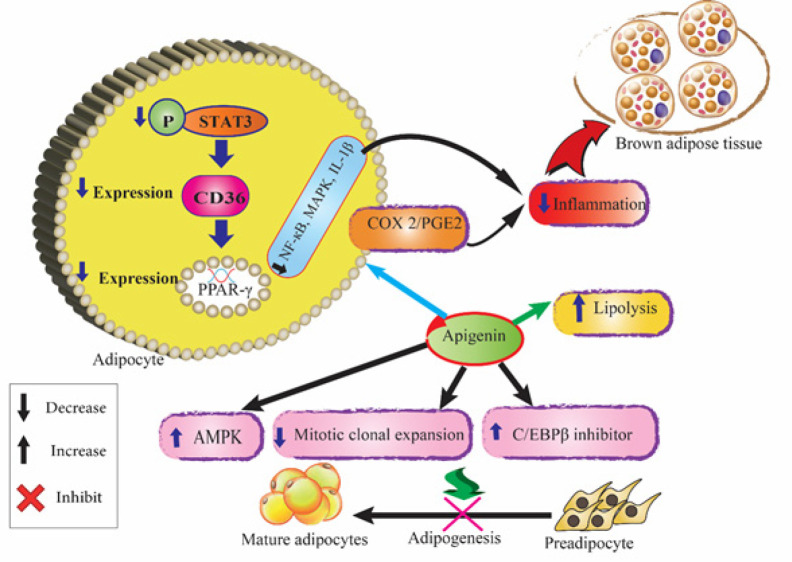
Schematic illustration of the effects of apigenin on obesity

Apigenin has been shown to potently suppress the differentiation of 3T3-L1 cells through reducing CCAAT/enhancer binding protein (C/EBP) α and PPARγ levels. Clonal expansion during the early stage of adipocyte differentiation was inhibited in apigenin-treated 3T3-L1 cells. Apigenin arrested cell cycle progression at the G0/G1 phase and significantly reduced cyclin D1 and cyclin-dependent kinase 4 expression. Therefore, apigenin can attenuate 3T3-L1 differentiation by suppressing the mitotic clonal expansion and the adipogenesis-linked factors and up-regulating the expression of multiple C/EBPβ inhibitors (29, 30). A study reported that apigenin (10, 20, and 40 µM) and emodin synergistically inhibited 3T3-L1 cell differentiation and lipase activity (31). Apigenin (50 μM) also decreased adipogenesis by down-regulating PPARγ function through AMPK activation in 3T3-L1 cells. This effect was 20 times stronger than that of 5-aminoimidazole-4-carboxamide ribonucleotide (an analog of AMP that serves to activate AMPK) indicating that apigenin possibly acts through modulating the activity of calcium/calmodulin-dependent protein kinase kinase-β (CaMKKβ) and thereby triggering the AMPK cascade in adipocytes (19). Feng *et al*. investigated obesity-mediated inflammation and metabolic problems in high-fat diet (HFD)-induced and ob/ob mice. Their results demonstrated that apigenin could bind to and modulate PPARγ activation, which in turn, blocked p65 translocation into nuclei by suppressing p65/PPARγ complex translocation into nuclei, thus, reducing NF-κB activation and increasing M2 macrophage polarization. Apigenin could significantly repolarize M1 macrophage into M2 and decrease the invasion of inflammatory cells into the liver and AT. Apigenin also reduced pro-inflammatory cytokine levels and alleviated inflammation. Moreover, it improved glucose resistance and attenuated the liver and muscular steatosis, and ALT, AST, total cholesterol (TC), and triglycerides (TG) levels without inducing significant weight gain and osteoporosis (32). Gentile *et al*. demonstrated that apigenin (10 mg/Kg/day) could attenuate the increase in body and epididymal fat weight, and reverse the changes in cholesterol, TG, and glucose levels in HFD mice. It lowered colonic levels of malondialdehyde (MDA), IL-1β, and IL-6, reduced eosinophil infiltration, substance P, and inducible nitric oxide synthase (iNOS) expression indicating its inhibitory effects on obesity-induced colonic inflammation. Apigenin counteracted the electrically evoked tachykininergic and nitrergic contractions which determine its protective properties against obesity-induced bowel dysmotility (33). It has been shown that the regulation of NAD^+^ metabolism especially increasing cellular NAD^+^ levels and possible activation of sirtuins would be a useful strategy to combat MetS. CD38 is the primary NAD^+^ase in mammals that hydrolyses NAD and modulates its homeostasis. Inhibition of CD38 leads to higher NAD^+^ levels and protection against obesity and MetS in mice. CD38 can modulate global protein acetylation by modifications in NAD^+^ levels which is reduced by apigenin. Moreover, apigenin can decrease the acetylation of p53 and p65, major players of the proinflammatory NF-κB complex. Apigenin treatment in obese mice also increased NAD^+^ levels and improved glucose and lipid homeostasis (34). Sun and Qu reported that dietary apigenin (0.04% (w/w) for 12 weeks) attenuated HFD-induced weight gain, glucose intolerance, and IR in C57BL/6 mice. Energy expenditure was enhanced without any change in energy intake, indicating that apigenin exerts its anti-obesity effects through increasing energy export. Moreover, apigenin could activate lipolytic enzymes (ATGL/FOXO1/SIRT1) without elevating cycling FFAs. FFA levels were decreased through up-regulation of FA oxidation AMPK/acetyl-CoA carboxylase (ACC), thermogenesis, AT browning enzymes, e.g., uncoupling protein (UCP-1, a mitochondrial protein abundantly expressed in brown adipocytes), and PGC-1α (a cofactor for PPARγ required for the adaptive thermogenic response to cold). In addition, inflammation markers of AT, NF-кB, and MAPK were decreased by apigenin treatment (35, 36). Inflammation-induced suppression of adipocyte browning was also induced by apigenin through activating the COX2/PGE2 pathway for stimulation of UCP-1 via EP4 activation (37). Researchers investigated the effect of various phenolic compounds including apigenin on fat accumulation in *Caenorhabditis elegans*. Apigenin at doses<500 µM has a potent lipid-reducing activity. Additionally, apigenin significantly up-regulated lipid metabolism-related genes such as lipl-5, which encodes for a lipase that breaks TAG to FFAs and glycerol thus exerting fat-reducing activity (38). Apigenin reduces the expression of CD36, a membrane glycoprotein that imports FA into the cells and plays a role in metabolic disorders. Overexpression of constitutive active STAT3 decreases the apigenin-suppressed adipogenesis indicating that the anti-visceral obesity effects of apigenin are associated with inhibition of STAT3/CD36 signaling axis (Figure 3) (39). 

Overall, apigenin exhibited anti-obesity properties via different mechanisms including suppression of mitotic clonal expansion, triggering the AMPK cascade in adipocytes, and up-regulating the expression of multiple C/EBPβ inhibitors to decrease adipocyte differentiation. Additionally, it acts by activating COX2/PGE2, reducing inflammation, and increasing brown AT as well as inhibiting the STAT3/CD36 signaling axis and suppressing adipogenesis.

***Effects of apigenin against diabetes***

Insulin resistance (IR) is an important aspect of T2DM and CVD and is closely associated with hyperglycemia, hyperinsulinemia, and hypertriglyceridemia which are common features of T2DM and MetS (40, 41). In HFD-induced obese mice, apigenin (0.005% w/w, p.o. for 16 weeks) attenuated IR and inflammation and significantly decreased levels of FBS, plasma insulin, and the homeostatic index of insulin resistance (HOMA-IR) (20). Protein tyrosine phosphatase 1B (PTP1B) is a negative modulator of the insulin and leptin signaling pathway that leads to IR. It is expressed in several tissues such as the liver, skeletal muscle, AT, and brain. Insulin binds to the extracellular α-subunit of its receptor and activates the insulin receptor through auto-phosphorylation (42). Further, tyrosine phosphorylation of insulin receptor substrates (IRS) leads to the activation of PI3K and Akt which in turn triggers the translocation of the glucose transporter 4 (GLUT4) to the cell surface to facilitate glucose transport. Moreover, PTP1B is responsible for de-phosphorylation of activated insulin receptors and further regulation of blood glucose. It also has a key role in regulating insulin sensitivity (43). Therefore, inhibition of PTP1B can be a promising therapeutic strategy to attenuate IR and T2DM. Ali *et al*. demonstrated that apigenin (6 and 12 µM) could inhibit PTP1B expression in a dose-dependent fashion. In addition, apigenin significantly elevated insulin-stimulated 2-NBDG (a fluorescent agent used for tracing glucose uptake into living cells) in insulin-resistant C2C12 cells (42). Apigenin (0.78 mg/kg; p.p. for 10 days) elevated serum insulin and thyroid hormone levels and reduced glucose levels and hepatic glucose-6-phospatase (G6Pase) activity. Apigenin could also attenuate the alloxan-induced increase in serum cholesterol and hepatic lipid peroxidation (LPO) and normalized the activity of anti-oxidant enzymes including catalase (CAT), superoxide dismutase (SOD), and glutathione (GSH) content (44). Apigenin (4 mg/kg/day for 7 days; IP) also significantly reduced the blood glucose levels in streptozotocin (STZ)-induced diabetic rats (45). In another study, Esmaeili *et al*. investigated the protective effects of apigenin (2 mM) on Stz-induced β-Cell failure in isolated rat pancreatic islets. Apigenin significantly elevated insulin levels by almost 50% compared to the control (46). Moreover, apigenin (5 μM) significantly reduced the intracellular ROS production, cellular DNA damage, and protein carbonylation, and attenuated the STZ-induced apoptosis in rat insulinoma cell lines (RINm5F pancreatic β cells) indicating its effects in the prevention of β cell dysfunction and T2DM (47). 

It is known that glucose toxicity can lead to progressive damage of pancreatic β-cell and the development of T2DM. OS plays an important role in glucose toxicity in β-cells. In a cellular model of 2-deoxy-D-ribose (dRib)-induced toxicity, apigenin (10µM) protected HIT-T15 pancreatic β-cells from oxidative cell damage by regulating the mitochondrial membrane potential (48). Apigenin (0.14 ± 0.02 𝜇M) has been shown to be a potent dipeptidyl peptidase IV (DPP-IV; an aminopeptidase that is involved in glucose metabolism). Moreover, analyses of computational modeling showed that apigenin docked to DPP-IV in a non-competitive manner. Hydrogen bonding was the main binding mode of apigenin with DPP-IV (49). 

A study investigated the effects of apigenin on the intracellular distribution of Forkhead box transcription factor O1 (FOXO1; a regulator of insulin signal transduction) to determine the state of insulin signaling. In U-2 OS (human osteosarcoma) cells, apigenin could trigger rapid intracellular translocation of FOXO1 which was reversed by insulin. The effects of apigenin on the expression of target genes were investigated in the HepG2 (human hepatoma) cell line. The mRNA expression of the gluconeogenic enzymes phosphoenolpyruvate carboxykinase (PEPCK) and G6Pase, lipogenic enzymes, fatty acid synthase (FAS), and ACC, were down-regulated by apigenin. The insulin-induced phosphorylation of PKB/AKT-, PRAS40-, p70S6K-, and S6- was reduced by apigenin indicating inhibition of the AKT signaling pathway. These results suggest that apigenin can exert its antidiabetic activity via decreasing gluconeogenic and lipogenic enzyme activity despite suppressing the PKB/AKT pathway (50). 

**Figure 4:**
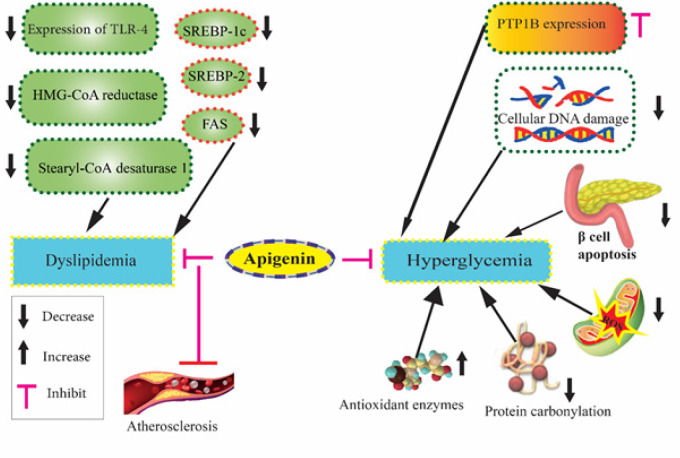
Schematic illustration of the effects of apigenin on hyperglycemia and hyperlipidemia

In STZ-induced diabetic rats, apigenin (1.5 mg/kg, IP; every alternate day for 28 days) could enhance GLUT4 translocation, down-regulate CD38 expression, control blood glucose levels, and normalize changes in hepatic phase I and phase II drug-metabolizing enzymes and anti-oxidant enzymes activities (51). Finally, in an *in vitro* study, apigenin at 0.5, 1, 1.5, and 2 mg/ml could inhibit α-glucosidase and α-amylase with IC_50_ values of 231.13 ± 5.35 and 287.53 ± 5.39 μg/ml, respectively, compared to those of the control and acarbose (IC_50 _= 996.02 ± 21.34 and 678.43 ± 16.52 μg/ml, respectively) (Figure 4) (52). 

Overall, a variety of mechanisms account for the hypoglycemic effects of apigenin as follows: inhibiting the expression of PTP1B, a negative modulator of the insulin and leptin signaling pathway; increasing the anti-oxidant enzymes and reducing ROS formation, DNA damage, and carbonylation of protein which are increased by T2DM; and preventing beta cell apoptosis in the pancreas.

***Effects of apigenin on dyslipidemia***

Dyslipidemia is a state that is defined by elevated levels of LDL, very low-density lipoproteins (VLDL) and TG, and reduced levels of HDL. Oxidation of LDL is considered a crucial step in the pathogenesis of atherosclerosis. Normally, insulin stimulates lipoprotein lipase (LPL; an enzyme that catalyzes the hydrolysis of TG) activity, but in the absence of insulin, LPL cannot be activated, leading to hyperglyceridemia and hypercholesterolemia. This state is closely related to elevated levels of pro-inflammatory cytokines such as TNF-α or IL-6 (53). 

Sterol regulatory element-binding proteins (SREBPs) are crucial transcriptional factors of lipid metabolism. SREBP-1a and SREBP-1c isoforms mainly activate the transcription of genes responsible for FA biosynthesis. SREBP-1c also is expressed in insulin-sensitive tissues including the liver and muscle (54). SREBP-2 is involved in cholesterol homeostasis by activating the transcription of sterol-regulated genes. In palmitate (PA)-induced hyperlipidemic HepG2 cells and HFD–fed mice, apigenin could significantly down-regulate SREBP-1c, SREBP-2, FAS, endoplasmic reticulum stress (ERS), stearyl-CoA desaturase 1, and HMG-CoA reductase, the key enzymes in the metabolism of FAs and cholesterol (33, 40). The inhibitory effects of apigenin on cholesterol production in HepG2 KCLB 88065 and MCF-7 KCLB 30022 cells were examined by Kim *et al*. The results showed that apigenin (35μM) suppressed the production of cholesterol in both HepG2 and MCF-7 cells (55). In a mouse model of hyperlipidemia, apigenin could significantly regulate blood lipids, decrease animal weight, and reduce total serum cholesterol (*P*=0.024), TG (*P*=0.031), and LDL (*P*=0.014). Apigenin also improved the blood lipid metabolism of the HFD mice by increasing cholesterol absorption and conversion and reducing the accumulation of intracellular cholesterol. Apigenin increased SOD activity and NO levels in H_2_O_2_-treated human umbilical venous endothelial cells (EA.hy926 cells) (*P*=0.043 and *P*=0.038, respectively) (56). Cholesterol efflux regulatory protein (CERP) is a key mediator of cholesterol efflux from macrophages, which is a protective process against atherogenesis. CERP serves to prevent foam cell formation by transporting excessive cholesterol out of lipid-loaded macrophages and maintaining homeostasis of lipids and cholesterol in macrophages (57). In RAW264.7 macrophages, apigenin (10, 20, 40 μM for 24 hr) could strongly elevate CERP expression through miR-33 repression in a dose-dependent manner. In macrophage-derived foam cells, apigenin treatment significantly enhanced CERP-mediated cholesterol efflux and decreased TC, free cholesterol (FC), and cholesteryl ester levels. In lipopolysaccharide-treated macrophages, apigenin also decreased the expression levels of Toll-like receptor-4 (TLR-4) which is involved in inflammation and atherosclerotic plaque formation (58). TLR signal transition takes place through a cytoplasmic adaptor protein, myeloid differentiation primary response gene 88 (MYD88), causing the activation of nuclear factor-κB (NF-κB) transcription factor and subsequent expression of inflammatory factors (59). Apigenin could decrease MyD88, p-IκB-α, and NF-κB p65 which indicates its protective effects against inflammation and atherosclerotic plaque formation. In harmony with *in vitro* results, apigenin could also increase CERP expression, reduce the contents of macrophages and smooth muscle cells in the atherosclerotic lesion, lower miR-33, TLR-4, and NF-κB p65 levels, improve plasma lipid profile (TC, TG, HDL-C, and LDL-C levels) and relieve inflammation, resulting in reduced atherosclerotic lesion size (56). In another experiment, apigenin dietary supplementation (50 ppm for 8 weeks) could modulate the total and serum non-HDL cholesterol and decreased the expressions of Sterol Regulatory Element Binding Transcription Factor 2 (SREBF2) mRNA, SREBP-2 protein, and HMG-CoA reductase in the livers of HFD mice. In addition, apigenin could activate AMPK indicating its inhibitory effects on the biosynthesis of cholesterol and attenuating HFD-induced hypercholesterolemia (60). In a hamster model of high-cholesterol diet-induced hypercholesterolemia, apigenin supplementation (60 and 300 ppm) could decrease the formation of aorta plaque by 30%. Apigenin at both concentrations increased hepatic LDL receptor expression by 1.5-fold and reduced non-HDL cholesterol levels by 40% as compared with those of the control group. Moreover, fecal elimination of cholesterol was elevated by 20% in the hamsters treated with 300 ppm apigenin. It was shown that suppressing the expression of the cholesterol transporter Niemann-Pick C1-like 1 (Npc1l1) in the intestinal mucosa could inhibit cholesterol absorption and facilitate its excretion (Figure 3) (61). 

Overall, the main mechanisms responsible for the hypolipidemic effects of apigenin include: decreasing dyslipidemia and subsequent atherosclerotic conditions via down-regulating SREBP-1c, SREBP-2, FAS, Stearyl-CoA desaturase 1, HMG-CoA reductase, and expression of TLR-4 which are involved in inflammation and atherosclerotic plaques formation.

***Effects of apigenin on steatohepatitis***

Feng *et al*. reported that apigenin (30 mg. kg/day; IP for 3 weeks) exerts a significant inhibitory influence on the expression of PPAR target genes producing the protein implicated in lipid metabolism. The apigenin-induced nuclear translocation of Nrf2 increased the expression of OS-related genes while decreasing the expression of lipid metabolism genes. These findings suggest that apigenin may support individuals with non-alcoholic steatohepatitis (NASH) by suppressing lipid metabolism and OS abnormalities via a new regulating mechanism of Nrf2 and PPAR (62). Apigenin (150–300 mg/kg/day p.o. for one month) has been shown to have a protective effect on alcohol-induced liver damage in alcoholic mice by modulating the expression of PPAR, which is involved in the synthesis of lipoproteins, and hepatic CYP2E1, which is associated with OS generation (63). Moreover, oral apigenin could protect against hepatocellular injury by reducing hepatic lipid deposition and inflammation. These protective effects could be partly attributed to the modulation of xanthine oxidase, which can regulate NLRP3 inflammasome activation and generation of inflammatory cytokines IL-1 and IL-18 (64). Apigenin (50, 100, and 200 mg/kg; p.o. for 7 days) reduced CCl4-induced liver injury by inhibiting the noncanonical NF-B pathway. Apigenin also preserved HepG2 cells from H_2_O_2_-induced injury and OS *in vitro* studies (65). 

***Effects of apigenin on hypertension***

An *in vivo* analysis found that apigenin (0.03, 0.05, and 0.11 g/kg; p.o. for 28 days) considerably decreased blood pressure (BP) in rats with spontaneous HR. Additionally, apigenin could down-regulate the level of endothelin-1 (ET-1) and angiotensin II (AngII), which is considered a key mechanism for its hypotensive effects (66). Apigenin (50–100 mg/kg; p.o. for 4 weeks) reduced the level of FFAs in serum and myocardial tissue, cardiomyocyte cross-sectional area, and improved HT, heart weight, and serum AngII in rats with renovascular HT. Additionally, apigenin reduced the amount of cardiac HIF-1 protein expression and increased the expression of PPARα, PDK-4, and CPT-1, while reducing the expression of PPARγ, GPAT, and GLUT-4. These results demonstrate that apigenin is capable of decreasing myocardial HT as well as impaired glucolipid metabolism in rat myocardial cells. This process is linked to HIF-1 inhibition, which raises the levels of CPT-1 and PDK-4 while decreasing the levels of GPAT and GLUT-4 (67). Apigenin could suppress the coupling of [3H] thymidine with DNA in a dose-dependent fashion in rat aortic smooth muscle cultured cells. In addition, apigenin could lower BP and induce endothelium-dependent relaxation as a consequence of the entry and release of NO, Ca^2+^, and cGMP (68). Pretreatment of rats with apigenin (intrarenal; 10–100 μM) could decrease contractions produced by KCl, thromboxane A2 analog U46619, phenylephrine, and vasopressin, in a dose-dependent way (IC_50_ values = 13.27–26.26 μM). Apigenin resulted in fast relaxation of arteries (RC_50_ =5.80–24.33 μM) while NOS inhibitors, chloride deficiency, Cl^¯ ^channel blockers, and voltage-gated potassium blockers could reverse this relaxant effect (69). Moreover, the impaired endothelial-dependent vasodilation of rat aorta induced by high glucose was reversed by apigenin through increased activity of endothelial iNOS and NO levels. In addition, apigenin inhibited the phosphorylation of protein kinase C βII (PKCβI) and reduced the activity of caspase-3, Bax expression, NF-μB phosphorylation, and apoptosis in endothelial cells with high glucose exposure (70). Apigenin (0.05g/ml) also induced vasodilation in pre-contracted rabbit aortic rings and frog heart via calcium channel and 1-β1- adrenoreceptor suppression (71). 

*In vitro*, apigenin also showed ACE activity with an IC_50_ value of 280 µM through cleavage of the chromophore-fluorophore labeled substrate dansyltriglycine by ACE I preparation from rabbit lung (EC 3.4.15.1) into dansylglycine (72). Apigenin also could reduce chronic HT induced by NG-nitro-L-arginine methyl ester (L-NAME) in rats and enhance NO-mediated and non-NO-mediated vasodilation. It also increased urinary nitrite excretion and reduced vascular damage to the extremities (73). Apigenin (25, 50, and 75 mg/kg; IP; 2 weeks) could balance heart rate and systolic and diastolic arterial pressures in an animal model of isoproterenol-induced cardiac damage. Apigenin was also found to be capable of ameliorating isoproterenol’s detrimental effects on left ventricular end-diastolic pressure (*P*˃0.001) and compensating for the increasing interval between the ST and RT in the ECG pattern. Apigenin treatment decreased the content of myocardial damage markers such as CK-MB isoenzyme and LDH (74). Apigenin (100 mg/kg/day for 42 days) was reported to reduce the size of sclerotic lesions in atherosclerosis-prone apolipoprotein E null mice. Apigenin could also promote apoptosis in oxidized LDL-loaded murine peritoneal macrophages. Apigenin’s proapoptotic actions were due to inhibiting phosphorylation of AKT at residue Ser473 of Akt1 binding site for apigenin, and reducing activation of plasminogen activator inhibitor 2 (PAI-2) (75). 

It is shown that aged mice have lower NO bioavailability, which causes impairing arterial endothelium-dependent dilation (EDD) and endothelial function. Apigenin (0.5 mg/ml, p.o.; 6 weeks) could improve EDD in older mice by boosting NO bioavailability, decreasing OS, and suppressing the production of free radicals. Apigenin also inhibited foam cell formation and reduced age-related aorta stiffening by regulating intrinsic wall stiffness and modifying extracellular matrix alterations and inflammation throughout aging (76). Apigenin infusion (20 μg/hr; p.o. 28 days) also had significant lowering effects on heart rate and mean arterial pressure, which was mediated through the reduction of NADPH oxidase-dependent ROS production and inflammatory response in the paraventricular nucleus (77). 

***Structure-activity relationship***

The pharmacological activities of bioactive molecules are highly associated with their structural features. The structure-activity relationship (SAR) analysis revealed that the presence of C5–OH, C7–OH, C2=C3, and C4=O functional groups in the apigenin and other flavonoids is associated with greater anti-inflammatory effect (78). Moreover, anti-oxidative, α-glucosidase, and α-amylase inhibitory activities of apigenin have been shown to be attributed to the presence of seven double-bonds in its two aromatic rings. In particular, double bonds between C-2 and C-3 are possibly crucial factors, and hydroxyls on rings A (C-7 and C-6) and B (C-4′) might serve as augmenters. Hydroxyl groups of apigenin are also suggested to be crucial moieties to chelate the zinc ions, thus inactivating the ACE activity (79). The hepatoprotective activity of apigenin which is through regulating the Nrf2/Keap1 anti-oxidant pathway, NF-κB signaling pathway, the release of pro-inflammatory cytokines, and apoptosis-related factors has been also shown to be attributed to the presence of C2=C3 double bond and the hydroxyl group of C4′ (80). 

***Bioavailability and pharmacokinetics of apigenin***

The identification of pharmacokinetic features of bioactive molecules can improve our knowledge of their bioavailability and help conduct appropriate human studies (17). Apigenin is found in food in its glycoside forms which are absorbed and distributed in aglycone form. It is classified as a biopharmaceutics classification system (BCS) class II drug due to its low solubility in water and lipid and high permeability and poor bioavailability after oral administration which results in being excreted unabsorbed or metabolized after absorption (18, 81). After oral administration, apigenin at 60 mg/kg could provide very low blood levels with approximate maximal circulating concentration (C_max_) of 1.33±0.24 μg/ml, an area under the curve (AUC_0–t_) of 11.76±1.52 μg⋅hr/ml after 0.5–2.5 hr (T_max_), with an elimination half-life (T_1/2_) of 2.52±0.56 hr (81, 82). Intestinal transport investigation revealed that apigenin can be absorbed in the whole intestine with the duodenum being the main absorption site. The absorption mechanism in ileum and colon segments is possibly passive transport with concentration-independent permeability behavior, while in duodenum and jejunum segments it might be both passive and active carrier-mediated transport (18). However, IV bolus injection of apigenin (20 mg/kg), resulted in a C_max_ of 10933.88 ± 1730.11 μg/L, AUC _(0−t)_ of 3211.54 ± 554 μg/L/h, and a systemic clearance of 6.12 ± 0.79 L/hr/kg. Noteworthy to mention that the volume of distribution at the terminal phase (Vz =15.75±11.73 L/kg) was far higher than the total body fluid (0.67 L/kg) in the rats indicating its extensive distribution into the tissues (83). It has been shown that apigenin has a relatively high elimination half-time of 91.8 hr compared with other major dietary flavonoids (e.g. luteolin and quercetin) which suggests its possible accumulation in tissues and the utility of apigenin-rich medications in the treatment of chronic diseases (84). *In vivo*, apigenin undergoes sulfation and glucuronidation and is found in blood and tissues mostly in the form of glucuronide, sulfate conjugates, or luteolin which are eliminated through urine and feces (85). Apigenin may involve in drug–drug interactions due to the inhibition of P-glycoprotein and CYP3A4. However, as it has synergistic effects with many antitumor medications and can improve the efficacy and bioavailability of co-treated drugs, a favorable drug–drug interaction profile is suggested (17). 

## Discussion

Apigenin as one of the most abundant flavonoids found in the plant kingdom could attenuate several ingredients of MetS. Apigenin possesses anti-obesity activity mainly by attenuating adipocyte differentiation by suppressing the mitotic clonal expansion and the adipogenesis-related factors, up-regulating the expression of multiple C/EBPβ inhibitors, and activating the COX2/PGE2 pathway for stimulation of UCP-1 via EP4 activation. Apigenin also acts by modulating the activity of CaMKKβ and thereby triggering the AMPK cascade in adipocytes as well as inhibition of STAT3/CD36 signaling axis. The effects of apigenin against hyperglycemia and IR can be exerted through mechanisms including inhibition of PTP1B expression, maintaining the activity of anti-oxidant enzymes, reducing the intracellular ROS production, cellular DNA damage and protein carbonylation, and attenuating β cell apoptosis and dysfunction through regulating the mitochondrial membrane potential. These mechanisms suggest that apigenin can alleviate obesity, IR, hyperglycemia, and the associated conditions. Apigenin could attenuate dyslipidemia and subsequent atherosclerotic conditions through down-regulating SREBP-1c, SREBP-2, FAS, stearyl-CoA desaturase 1, HMG-CoA reductase, TLR-4, Npc1l1, MyD88, p-IκB-α, and NF-κB p65 which are involved in inflammation and atherosclerotic plaques formation. 

As mentioned before, apigenin in its aglycone forms have poor oral bioavailability. Novel drug delivery systems such as liposomes, neosomes, and nano-emulsions would be a great help to enhance the potency and oral bioavailability of apigenin (86). 

Recently, numerous clinical trials have been conducted to evaluate the effects of several medicinal plants containing high amounts of apigenin; however, to our knowledge, there is no human study showing the efficacy, safe dosage, pharmacokinetics features, and possible side effects of apigenin in human subjects. Therefore, designing clinical trials to study the efficacy of apigenin in the treatment of MetS and its ingredients would be necessary to provide proper information to support the results of *in vitro* and experimental studies. Altogether, apigenin would be a promising candidate for improving MetS and its components.

## Conclusion

Dietary bioactive compounds have been shown to tackle a variety of diseases, e.g., diabetes, dyslipidemia, hypertension, obesity, etc. Hereto, we found that apigenin, a flavonoid present in several vegetables and fruits, possesses protective effects against various components of MetS. Our findings may have implications for developing new medications to improve MetS. Apigenin is involved in the pathogenesis of MetS through several molecular pathways. However, future cellular and animal investigations is warranted in order to shed light on other signaling pathways that account for the protective effects of apigenin against MetS. Moreover, conducting quality human trials to elucidate the optimum human doses, bioavailability, drug interactions, unwanted side effects, and pharmacokinetic characteristics are needed. 

## Authors’ Contributions

B J and Z S conceived the study, wrote the original draft, and reviewed and edited the manuscript. 

## Conflicts of Interest

There are no conflicts of interest.
